# Valorization of Local Agricultural Byproducts for the Development of Functional Oat-Based Milk Formulations

**DOI:** 10.3390/foods14081436

**Published:** 2025-04-21

**Authors:** Diana De Santis, Riccardo Frisoni, Alice Rossi, Serena Ferri, Margherita Modesti

**Affiliations:** Department for Innovation in Biological, Agro-Food and Forest Systems (DIBAF), University of Tuscia, Via San Camillo De Lellis snc, 01100 Viterbo, Italy; desdiana@unitus.it (D.D.S.); riccardo.frisoni@unitus.it (R.F.); aliceros1101@gmail.com (A.R.); serenaferri@unitus.it (S.F.)

**Keywords:** oat, local variety, olive leaves, raspberries leaves, raspberries powders, byproducts

## Abstract

Background: Consumer demand for plant-based milk alternatives, particularly oat-based milk, has increased due to perceived health benefits and environmental sustainability. However, challenges remain in improving their nutritional profile and physical stability while promoting the use of local agricultural resources and reducing food waste. Methods: This study developed and evaluated fortified oat-based milk formulations using locally sourced oats cultivated in central Italy. Two valorization strategies were tested: (i) the addition of raspberry powder derived from juice processing byproducts and (ii) the substitution of water with infusions of raspberry and olive leaves. The nutritional composition, antioxidant activity, physical stability, and sensory properties were assessed. Results: Replacing water with leaf infusions significantly increased total polyphenol content (up to 688 mg GAE/100 g DW) and antioxidant activity but compromised physical stability, resulting in higher separation indexes during refrigerated storage. Conversely, adding raspberry powder moderately enhanced antioxidant properties while maintaining emulsion stability. Sensory evaluation showed that enriched formulations reduced undesirable attributes (e.g., floury and cereal notes), although higher concentrations of leaf infusions increased astringency and bitterness. Conclusions: The fortification of oat-based milk with locally sourced raspberry powders and leaf infusions effectively enhances its nutritional and antioxidant properties while influencing its physical and sensory characteristics. This strategy supports the valorization of local agricultural byproducts and promotes the development of sustainable, functional plant-based beverages.

## 1. Introduction

In recent years, consumer demand for plant-based beverages, also known as plant-based milks, has significantly increased. This increase can be attributed to various factors, such as health and environmental issues, lactose intolerance, and a growing trend toward flexitarian diets [1]. The plant-based milk market, valued at USD 15.9 billion in 2023, is projected to grow at a compound annual growth rate (CAGR) of 11.6% from 2024 to 2032 [2]. Additionally, sales of oat milk surged by over USD 60 million, marking an annual increase of nearly 700% from 2018 to 2019 [3]. Plant-based milks are water-soluble extracts that result in an emulsion resembling cow’s milk. Among the different options, beverages based on legumes (mostly soybeans), cereals (like rice and oats), and nuts (such as almonds and hazelnuts) are the most popular [4]. Generally, plant sources (cereals and legumes) are perceived as functional and nutraceutical foods due to their health-promoting components like vitamins, fibers, minerals, and antioxidants [5]. As such, most consumers today believe that including plant-based ingredients in their diet is essential [5].

Oat milk was developed in the 1990s by Swedish researchers to offer an environmentally friendly alternative to dairy milk [6]. It is considered a basic plant-based nutritional drink and a component of a healthy lifestyle [7]. Oat milk has gained significant traction as a popular cereal milk alternative and is now a major player in the plant-based milk market [8]. It has a smooth, milk-like texture, a sweet flavor, and whitish color. Oat milk production has a lower environmental impact in terms of lower greenhouse gas emissions, land use, and water use compared to cow’s milk [9].

Oat milk is particularly renowned for its rich nutritional profile, which comprises dietary fiber (especially β-glucan), proteins, and anti-inflammatory and antioxidant substances such as avenanthramides, avenacosides, and phenols [10]. Unlike cow’s milk, oat-based beverages are lactose- and cholesterol-free [1]. Like other plant-based milks, oat milk is a water extract of oats in which water represents about 90% of the final product. It is typically produced through a process of maceration, grinding, and filtration, which allows for the extraction of oat components in water.

Oat milk, while very popular, still faces several challenges related to its nutritional and sensory profile. First of all, during the production process, some of the oat nutrients are diminished. As such, to achieve higher nutritional value, oat milk is often fortified with nutrients such as vitamins, minerals, and proteins [11]. These fortified oat-based milks appeal to those seeking lactose-free options while providing comparable nutritional benefits. Consequently, fortified plant-based milks are becoming increasingly popular among health-conscious individuals, flexitarians, and those with dietary restrictions. Moreover, oat milk tends to sediment easily, which affects its texture and mouthfeel. This is a common problem during storage that decreases the product’s appeal [12]. Moreover, common negative sensory attributes associated with oat milk include “sandy” and “grainy” textures [13]. These attributes can significantly reduce consumer liking and acceptance. As such, optimization of processing techniques is often necessary to enhance the stability and sensory profile of oat milk [4].

In recent years, growing attention has been given to the use of agricultural byproducts as functional ingredients in food formulations. These byproducts—such as fruit pomace, peels, and leaves—are typically discarded despite being rich in valuable bioactive compounds, including polyphenols, fibers, and antioxidants [14,15]. Their incorporation into plant-based beverages contributes to improving the nutritional and functional profile of the product, supporting waste reduction, and promoting more sustainable food systems. In the context of plant-based milk production, the use of such byproducts offers an opportunity to address current challenges related to fortification and environmental impact in a single approach [16,17].

Another emerging trend is the adoption of environmentally friendly production methods. With growing consumer awareness of environmental issues [18], manufacturers are focusing on reducing their carbon footprint and minimizing their environmental impacts. This includes sourcing raw materials from sustainable agriculture, optimizing water and energy usage, using renewable energy sources, and implementing eco-friendly packaging solutions to reduce waste [2].

In this context, the focus of the present study is the production of oat-based milk (OBM) using local oats (cultivated in central Italy, Viterbo province) with the aim of promoting short food chains and supporting regional agricultural production. Furthermore, this research explores a novel approach to nutritional fortification through the valorization of agricultural byproducts, in line with the principles of the circular economy. Specifically, the oat milk was enriched with raspberry powder, obtained from the solid residues (seeds and skins) of raspberry juice production, and infusions of raspberry and olive leaves, both collected from local agro-industrial processes. These byproducts, often discarded as waste, are known to be rich in polyphenols, flavonoids, and antioxidants, and their integration into food matrices offers a sustainable and functional enrichment strategy. The proposed formulation has the dual goals of increasing the nutritional and functional profile of the final product while contributing to the reduction of food waste and the valorization of locally sourced resources.

## 2. Materials and Methods

### 2.1. Reagents

Ethanol (≥99.8%), methanol (≥99.9%), sodium carbonate (Na_2_CO_3_, ≥99.5%), potassium persulfate (K_2_S_2_O_8_, ≥99.0%), and Folin–Ciocalteu phenol reagent were all purchased from Merck (Darmstadt, Germany). These reagents were used for the extraction of antioxidant compounds, the Folin–Ciocalteu assay, and for the activation of the ABTS radical solution. Gallic acid monohydrate (≥98%), Trolox (≥97%), 2,2-diphenyl-1-picrylhydrazyl (DPPH, ≥95%), and 2,2′-azino-bis(3-ethylbenzothiazoline-6-sulfonic acid) diammonium salt (ABTS, ≥98%) were obtained from Sigma-Aldrich (St. Louis, MO, USA) and used as standards and reagents for the quantification of total phenolic content and antioxidant activity. Ultrapure water was produced using a Milli-Q purification system (Merck Millipore, Darmstadt, Germany) and was used for all aqueous extractions, dilutions, and analytical procedures. The enzymes α-amylase (ENDOZYM Alphamyl) and protease (ENDOZYM Proteasi NP), both food-grade, were supplied by AEB Group S.p.A. (San Polo di Piave, Treviso, Italy). These enzymes were used during oat-based milk production to promote starch and protein hydrolysis, respectively. Unless otherwise specified, all reagents were used as received without further purification.

### 2.2. Oat Material and Characterization

Naked oat cultivar (*Avena nuda*, cv Patrik) was cultivated and collected at Azienda Agricola Fornovecchino (str, Km. 4/800, 01100 Ombrone Viterbo, Italy, 42.49627, 12.09342). Raw oat grains were first subjected to proximate analysis. Specifically, dry matter, moisture, and ash content were determined using standard American Association of Cereal Chemists (AACC) methods [19]. Crude protein content was calculated from total nitrogen content, which was measured using an elemental analyzer (Vario Macro Cube CHNOS Elemental Analyzer, Elementar Analysensysteme GmbH, Elementar-Straße 1, 63505 Langenselbold, Germany). The nitrogen content was then multiplied by a conversion factor of 5.83 to express the results as crude protein [20]. After the analysis, the oat grains were used for oat milk production.

### 2.3. Oat-Based Milk (OBM) Production

**Figure 1:**
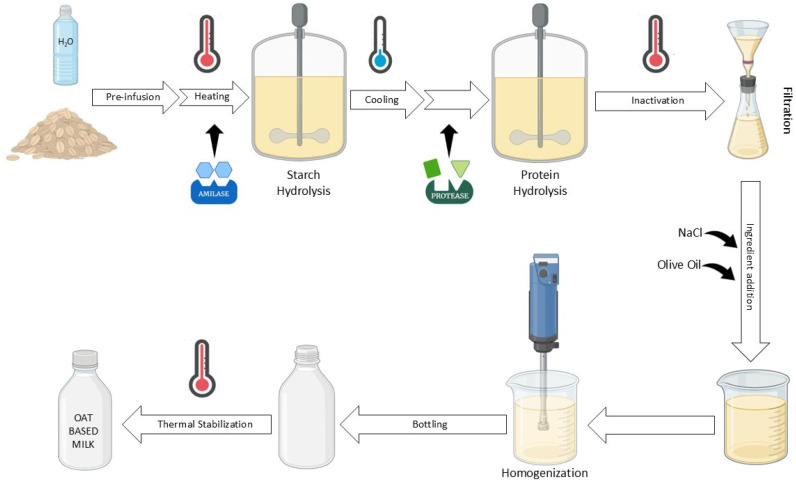
Workflow of OBM production.

The manufacturing process is summarized in Figure 1. Whole oat grains were coarsely ground and soaked in water (1:6 *w*/*w*) at room temperature for 15 min. Then, 0.5% (*w*/*w*) of α-amylase (ENDOZYM Alphamyl, AEB Group spa, San Polo, Italy) was added, and the ideal hydrolase temperature (90 ± 2 °C) was reached and maintained for 30 min. The mixture was cooled to 60 ± 1.5 °C, and 0.014% *w*/*w* of protease (ENDOZYM Proteasi NP, AEB Group spa, San Polo, Italy) was added and left to operate for 1 h. After 1 h, the enzymes were inactivated at 95 ± 0.5 °C for 5 min, and then the sample was filtered sequentially through a domestic strainer, a 200-mesh cloth, and a 400-mesh cloth. Then, 0.1% *w*/*w* of NaCl and 0.5% of extra virgin olive oil were added. The mixture was homogenized at 20,000 rpm for 3 min using a rotor-stator homogenizer (IKA T 25 digital ULTRA-TURRAX, KA, Staufen, Germany). Finally, the sample was thermally stabilized for 15 min at 90 ± 1.5 °C.

This protocol has been used as the standard procedure to produce control oat milk (OM) and enriched oat milks. However, for the production of enriched oat milk, the following additional procedures have been used. For each thesis, three separate replicates were produced, each with a final volume of 600 mL.

### 2.4. Enrichment of Oat-Based Milk with Raspberry Powder

All OBMs have been produced using the standard protocol described above. Raspberry powder (RP, 1.5% and 3% *w*/*w* of oats) was added along with ground oat grains at the beginning of the soaking phase, replacing an equivalent amount of oats to maintain the overall ingredient proportion (hereafter referred to as RP 1.5 and RP 3). The raspberry powder was obtained from the solid residues (comprising seeds and skins) remaining after juice extraction from *Rubus idaeus* L. cv. Heritage. These residues were oven-dried using an electric oven (Combi Plus 8 × 2) at 48 ± 2 °C for 16 h until a final moisture content of 10.87 ± 0.032% was reached. The dried material was then milled using a wood mill (KoMo GmbH & Co. KG Penningdörfl 6, 6361 Hopfgarten, Austria) and subsequently passed through a stainless-steel sieve with a mesh size of 300 μm (M.R.S. Scientific Ltd., Pilot Cl, Fulmar Way, Wickford SS11 8YW, UK) [21,22].

### 2.5. Substitution of Water with Infusions

The infusions of raspberry leaves (RL) and olive leaves (OL) were used to completely replace the water during the production of oat-based milks (OBMs). Both raspberry leaves (*Rubus idaeus* L. cv. Heritage) and olive leaves (*Olea europaea* L. cv. Canino) were collected from local producers (namely, raspberry juice and olive oil producers) and oven-dried (electric oven Combi plus 8 × 2) at 50 ± 2 °C for about 16 h. The plant material was chopped and mixed in a 1:75 ratio with low-mineral-content water, which had been previously heated to 90 ± 2 °C. The mixture was maintained at 90 ± 2 °C on a magnetic induction plate for 10 min. After infusion, the liquid was filtered through a 200-mesh food-grade sieve, left to cool, and then used for oat milk production.

### 2.6. Separation Index

The stability of OBM samples was measured using the method of Li et al. [23]. OBMs were placed in 50 mL graduated cylinders and stored at 4 ± 1.5 °C. The separation index (*SI*) was measured at 0, 1, 3, 5, and 7 days of cold storage and calculated using the following equation:

$$SI\;\left(\%\right)=\left(1-\frac{Hl}{Ht}*100\right)$$

where *Ht* is the total height of the sample in the bottle (mm), and *Hl* is the height of the lower phase (mm).

### 2.7. Proximal Analyses

For the measurement of moisture content (MC), 3 g of OBM samples were placed in an oven at 105 °C until a constant weight was reached. Dry matter (DM) was calculated using the following equation:

$$DM\;\left(\%\right)=\frac{Wf\;\left(g\right)}{Wi\;\left(g\right)}*100$$

where *Wf* is the final weight, and *Wi* is the initial weight.

*MC* was determined using the difference as follows:

$$MC\;\left(\%\right)=100-DW\left(\%\right)$$

The dried samples were incinerated in a muffle furnace at 550 °C overnight until white-gray ashes were obtained. The percentage ash content (*AC*) was calculated using the following formula:

$$Ash\;\left(\%\right)=\frac{Ashes\;\left(g\right)}{Dried\;sample\;\left(g\right)}*100$$

The mass yield (*MY*) percentage of the OBMs was estimated using an analytical balance by relating the weight of the final product to the total weight of the ingredients, according to the following formula:

$$MY\;\left(\%\right)=\frac{oat\;milk\;\left(g\right)}{\;oat\;grains\;\left(g\right)+water\;\left(g\right)}*100$$

### 2.8. Preparation of Extracts for the Analyses

To prepare the extract, 0.5 g of each OBM sample was mixed with 9.5 mL of an 80% ethanol solution and stirred to ensure complete homogenization. After mixing for 30 min, the samples were centrifuged at 8500 rpm for 20 min at 4 ± 0.5 °C. The resulting extracts were then filtered using Whatman Chr 1 filters. The extracts were either used immediately for analyses or stored at −20 °C. Extraction was performed in triplicate for each sample.

To extract bioactive compounds from raspberry powder and dried leaves, 1 g of each powdered sample was mixed with 10 mL of 80% methanol. The mixture was subjected to ultrasonic-assisted extraction using an ultrasonic bath (model DU32, Argo Lab, Via della Meccanica, 25, 41012 Carpi, MO, Italy) operated on ice for 15 min at a working frequency of 40 kHz and a maximum input power of 120 W. After sonication, the samples were centrifuged at 4100 rpm for 20 min using a refrigerated centrifuge (NEYA 16R, REMI ELEKTROTECHNIK LTD. Survey 65/1, Valiv Village, Vasai (East), Palghar-401 208, India) [21,22]. The resulting supernatants were collected and stored at −20 °C until analysis. All extractions were carried out in triplicate for each sample.

### 2.9. Total Phenolic Content

Total phenolic content (TPC) in the extracts was determined using the Folin–Ciocalteu method [24], with slight modifications. In brief, 1580 μL of deionized water was combined with 20 μL of sample extract and 100 μL of Folin–Ciocalteu reagent. The mixture was left to react in the dark at room temperature for 5 min. Subsequently, 300 μL of a 20% sodium carbonate (Na_2_CO_3_) solution was added, and the reaction mixture was incubated at 40 °C for 30 min. Absorbance was then measured at 765 nm using a UV–VIS spectrophotometer (Lambda 850+, Perkin Elmer, 940 Winter Street Waltham, MA 02451, USA). The phenolic content was quantified by interpolating the absorbance values on a gallic acid calibration curve (R^2^ = 0.9996), and the results were expressed as milligrams of gallic acid equivalents per gram of dry weight (mg GAE/g dw). All analyses were performed in triplicate on three independent OBM samples for each formulation.

### 2.10. Antioxidant Activity

Antioxidant activity was assessed using two widely established methods: the 2,2-diphenyl-1-picrylhydrazyl (DPPH) and the 2,2′-azino-bis(3-ethylbenzothiazoline-6-sulfonic acid) (ABTS) radical scavenging assays. The DPPH assay was carried out following the protocol of Xiao et al. [25] with minor modifications. Briefly, 5000 μL of sample extract was mixed with 250 μL of a 0.5 mM DPPH methanolic solution (final DPPH concentration: 0.1 mM) and 500 μL of methanol. The mixture was vigorously shaken and incubated in the dark at room temperature for 60 min. The absorbance was then measured at 517 nm using a UV–VIS spectrophotometer (Lambda 850+, Perkin Elmer, 940 Winter Street Waltham, MA 02451, USA), with methanol as the blank. A standard calibration curve was prepared using Trolox (6-hydroxy-2,5,7,8-tetramethylchroman-2-carboxylic acid, Sigma-Aldrich) at concentrations ranging from 3 to 27 µg/mL (R^2^ = 0.9967). Antioxidant capacity was expressed as milligrams of Trolox equivalents per gram of dry sample (mg TE/g dw). The ABTS radical scavenging activity was determined based on the method described by Re et al. [26] with slight adjustments. A 7 mM ABTS stock solution was reacted with 140 mM potassium persulfate (final concentration 2.45 mM) and kept in the dark at room temperature for 14 h to allow for radical generation. Prior to analysis, the ABTS⁺ solution was diluted with ethanol to obtain an absorbance of 0.7 ± 0.02 at 734 nm. For each sample, 20 μL of extract was added to 980 μL of the diluted ABTS solution and allowed to react for 60 min. Absorbance was then recorded at 734 nm against a blank. Antioxidant activity was expressed as mg of Trolox equivalents per gram of dry sample (mg TE/g dw), using a calibration curve ranging from 3 to 450 µg/mL (R^2^ = 0.9953). All measurements were performed in triplicate on three different OBM replicates per formulation.

### 2.11. Sensory Evaluation

To develop the lexicon, eight sensory judges (three men and five women, aged between 26 and 35) were recruited from the university department staff (DIBAF—University of Tuscia, Viterbo, Italy) and trained and monitored according to ISO standards no. 8586-1 [27] and 8586-2 [28]. The generation of descriptors for the OBM samples was performed in individual panel booths of a sensory laboratory conforming to ISO norm no. 8589 [29]. Prior to the sensory evaluation, two preliminary sessions were conducted to familiarize the assessors with the characteristics of the oat-based milk samples. The development of the sensory lexicon was carried out over three dedicated sessions, following the guidelines of ISO standard 13299 [30]. During these sessions, each panelist independently assessed the samples, which were presented in monadic sequential order, in order to identify the most representative attributes. All descriptors proposed by the panelists were collected and subsequently refined by eliminating non-relevant terms and merging synonyms or overlapping descriptors that referred to the same sensory characteristics. As a result, the final lexicon was reduced to 15 key terms, as reported in Table 1. To complement the descriptive analysis, an overall quality score was also assigned to each sample based on the absence of defects and the overall balance among the evaluated sensory attributes [31].

The descriptive sensory analysis was conducted by the same trained panel that was previously involved in the lexicon development. Each judge received a single portion of each of the five OBM samples. The samples were served in odorless plastic cups, labeled with three-digit random codes, and accompanied by an evaluation form and a glass of moderately mineralized water for palate cleansing. The evaluation form was structured in two sections: the upper section collected personal information and provided instructions on how to conduct the test, while the lower section listed the selected sensory attributes, whose intensity was rated by the panelists using a five-point scale.

### 2.12. Statistical Analysis

All chemical–analytical procedures were performed on three different extracts for each OBM preparation. All analytical data were subjected to the Bartlett and Shapiro–Wilk tests to verify normality and homogeneity of variances before performing one-way ANOVA and Tukey’s test for multiple comparisons at *p* < 0.05. Statistical analysis was performed by using GraphPad Prism (© 2024 GraphPad Software, 225 Franklin St FL 26 Boston, MA, 02110-2853 USA). Sensory product characterization was conducted using XLSTAT^®^ statistical software Premium version (2020.1.3; Addinsoft, NY, USA; https://www.xlstat.com).

## 3. Results and Discussion

### 3.1. Raw Material Characterization

The oat used in this study had a dry matter content of 94.85 ± 1.62%, a moisture content of 5.15 ± 1.62%, and a protein content of 9.19 ± 0.19%.

Table 2 shows the characterization of raw biological materials used for the fortification of oat-based milk. Olive leaves showed the highest DW (94.93%) and the lowest MC (5.07%), while raspberry powder had the highest MC (6.33%); however, none of these results were statistically significant. Raspberry leaves had the highest TPC (3650 mg GAE/g dw), suggesting that they are the richest source of polyphenols, known for their health-promoting properties, including antioxidant effects. Consequently, antioxidant activity, assessed through DPPH and ABTS assays, confirmed the higher bioactive activity of raspberry leaves, where the highest DPPH (5.67 TAEC mg/g dw) and ABTS (9.62 TE mg/g dw) inhibition values were observed. Olive leaves demonstrated medium antioxidant capacity (5.57 TAEC mg/g dw, 9.54 TE mg/g dw), while raspberry powder exhibited the lowest antioxidant potential. This variation is in agreement with previous studies that demonstrated that in different plants, leaf extracts generally have higher polyphenol and antioxidant activities compared to fruit extracts [32,33,34]. In our case, this is likely due to higher processing-related degradation of phenolic compounds occurring in raspberry byproducts during juice production, which may have resulted in the loss of significant amounts of polyphenols and their associated antioxidant properties. Thus, the thermal and mechanical processes involved in juice extraction can break down phenolic compounds, contributing to the lower bioactivity observed in fruit powders. The higher phenolic content and antioxidant activity of raspberry and olive leaves suggest their potential as functional ingredients for enriching oat-based milk with bioactive compounds. On the other hand, the lower phenolic content and antioxidant capacity of raspberry powder highlight that it may contribute more to the sensory profile, such as color and flavor, rather than bioactivity.

Significant differences were observed in the olive and raspberry leaf infusion extracts. The infusions presented an important different profile compared to their respective raw materials. Specifically, raspberry leaf infusion showed the highest TPC content, suggesting a significant extraction of polyphenols into the aqueous phase. This result is also confirmed by the higher antioxidant capacity measured by DPPH (21.01 TAEC mg/g dw) and ABTS (7.20 TE mg/g dw) assays. The higher phenolic content and antioxidant activity observed in raspberry leaf infusion further reinforce its potential as a functional ingredient for oat-based milk fortification. Similarly, olive leaf infusion showed a significant increase in DPPH (16.91 TAEC mg/g dw). However, its TPC (2049 mg GAE/g dw) was significantly lower than that of raspberry leaf.

### 3.2. Oat-Based Milks: Proximal Analysis and Stability

The different OBM formulations showed similar values in the proximate analysis, where no statistical differences were observed in terms of DM and MC (Table 3).

**Figure 2:**
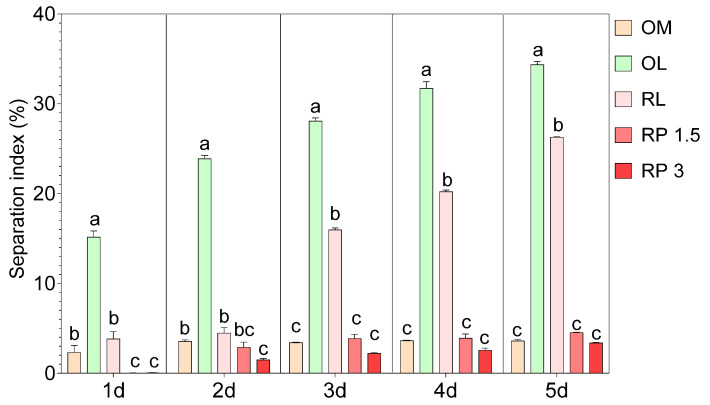
Separation index (%) mesaured at 1, 2, 3, 4, and 5 days of cold storage in different oat-based milk: OM = control oat-based milk, OL = oat-based milk with the substitution of water with olive leaf extract, RL = oat-based milk with the substitution of water with raspberry leaf extract, RP 1.5 = oat-based milk enriched with 1.5% raspberry powder, and RP 3 = oat-based milk enriched with 3% raspberry powder. Data are the mean of three biological replicates ± standard deviation. Different letters represent significant differences (*p* ≤ 0.05) according to one-way ANOVA and Tukey’s post hoc test.

On the other hand, the SI revealed important differences in the stability of the OBM formulations during 5 days of refrigerated storage. Specifically, the control sample (OM) exhibited a low separation index over time, ranging from 2.31% to 3.61% (Figure 2). The use of olive leaf infusion (OL) significantly increased the SI; on the first day of cold storage, the OL sample already had an SI of 15.16% and reached 34.37% by the final stage. Similarly, the sample with raspberry leaf infusion (RL) showed an increase in the SI over time, reaching 26.29%, although the initial values were lower than those observed for the OL, indicating slightly better stability. The fortification of OBM with raspberry powder resulted in an SI similar to that observed in the control OM and well below the OL and RL samples. Specifically, in the RP 1.5 sample, the separation index remained below 4.53% throughout the measurement period, and in the RP 3 sample, it was even lower, reaching a maximum of 3.38%. The production of OBM, although one of the most important plant-based milk alternatives, still faces significant challenges, mainly in emulsion solid–liquid separation [35]. The stability of OBM is affected by several factors. For instance, a higher protein content in oat kernels is negatively correlated with the stability of OBM, leading to increased creaming and a higher instability index [12]. On the other hand, starch content is positively correlated with stability and reduces sedimentation [12]. Moreover, the use of enzymes (protease and amylase) and heat treatments, such as blanching and microwaving, can significantly improve the stability of OBM. In our case, considering the low separation index observed in the OM control, it is evident that the optimized process is effective in producing a stable OBM. However, substituting water with the infusion, which is expected to increase the bioactivity of the OBM, significantly decreased its stability, thus highlighting that while the replacement of water with leaf infusions may introduce beneficial bioactive compounds, it also worsens physical stability. This replacement of water with leaf infusions might interfere with the stabilization mechanisms of the emulsion. This could be attributed to the introduction of polyphenols or other hydrophilic compounds that alter the interfacial properties between solid particles and the liquid phase, promoting separation. On the other hand, the addition of raspberry powders demonstrated a stabilizing effect. The stability effect of raspberry powders may be attributable to the presence of fibers and pectin. For instance, Cheng et al. [36] demonstrated that incorporating citrus fiber into acidic milk beverages reduces particle aggregation and sedimentation. Moreover, the addition of pectin enhanced the stability of acidified milk [37]. However, to the best of our knowledge, there are no other studies reporting the use of fruit powder or the substitution of water with leaf infusion for OBM production. Hence, most studies report fortification with β-glucan [38], proteins [13], vitamins, and minerals [39]. Therefore, it is difficult to draw definitive conclusions on why the stability changes so significantly.

### 3.3. Oat-Based Milks: Polyphenols and Antioxidants

Results obtained for TPC showed a significant impact of the different formulations (Table 4). Specifically, in the oat-based milk where water was replaced with leaf infusion, TPC was significantly higher. The highest TPC content was determined in the sample with raspberry leaf infusion (688 ± 77.99), in accordance with the highest polyphenol content observed in the infusion itself (Table 1). On the other hand, the addition of raspberry powder did not increase the TPC level, which remained similar to the control OM, likely due to the high dilution of the powder in the final product. Control OM presents a modest amount of phenols. As previously reported, oat is known for its important antioxidant capacity, mainly due to the high content of bioactive compounds such as polyphenols, avenanthramides, and β-glucans [40]. In particular, avenanthramides are a phenolic compound unique to oats, which have strong antioxidant and anti-inflammatory effects [40].

In terms of antioxidant capacity, as measured by the DPPH and ABTS assays, the highest values were observed in the oat-based milk with raspberry leaf infusion (RL), followed by the milk with olive leaf infusion (OL). These results align perfectly with the phenolic content observed in these samples. Raspberry leaves contain a substantial amount of polyphenols, which are crucial for their antioxidant activity [41]. These polyphenols include tannins, flavonoids, and phenolcarboxylic acids. When compared to other parts of the raspberry plant, such as the fruit and stems, the leaves generally have higher antioxidant activities. This is attributed to the higher concentration of polyphenols in the leaves [42,43]. Similarly, olive leaves are known to be a rich source of antioxidants and phenolic compounds, such as oleuropein, flavonoids, and other polyphenols [44]. These properties have been increasingly utilized to transform what was once considered a byproduct (i.e., leaves) into important sources of bioactive compounds.

OM showed the lowest ABTS and DPPH values, while RP samples, although they exhibited a phenolic content similar to that of the OM sample, showed slightly higher ABTS and DPPH activity. This may indicate that other compounds in the fruit powders contribute to the antioxidant activity. For instance, the antioxidant activity of raspberries is primarily attributed to their rich content of polyphenols, but also to ascorbic acid [45]. The ascorbic acid in raspberries ranges between 5–40 mg/100 g, contributing significantly to the overall antioxidant capacity of raspberry fruits [45].

Overall, the substitution of water with oat-based milk infused with raspberries and olive leaves strongly increases bioactive and antioxidant compounds. However, we must highlight that this replacement significantly increases the instability of the OBMs. Therefore, the balance between bioactive enrichment and physical stability needs to be carefully considered. On the other hand, the inclusion of RP at different concentrations slightly increases antioxidant capacity while maintaining physical stability.

### 3.4. Oat-Based Milks: Sensory Analysis

The collected data were processed using XLSTAT (Lumivero), applying a product characterization test to identify the most effective descriptors for discriminating between the samples evaluated by the sensory panel. The application of an ANOVA model to each descriptor determined whether the judges’ scores significantly differentiated the products. The model evaluated both the “product” and “judge” effects for each attribute, returning coefficients and *p*-values for every product-descriptor combination.

Descriptors with *p*-values lower than 0.05 were considered significant discriminators. As shown in Table 5, the most discriminating attributes were oat aroma (OA), flour (FL), cooked starch (CS), cereal (CE), and sourness (SO). Conversely, persistence (PE) and viscosity (VI) did not differ significantly among the samples.

Table 6 summarizes the adjusted means of each descriptor across the five samples. Products enriched with raspberry powder or leaf infusions showed a marked reduction in the intensity of negative attributes such as flour, cereal, oat, and cooked starch aromas. At the same time, enriched samples displayed lower sweetness and increased acidity and astringency compared to the control oat milk. The sensory attributes chosen for the characterization of the products in the OBM integrated with 1.5% raspberry powder have a medium intensity that gives a good balance to the flavor, attenuating the floury perception without enhancing the acidic and bitter notes. A slight astringency contributes only a negative note. The simultaneous tasting of the five products highlighted differences in the intensity of attributes that may not necessarily be negative for the consumer. However, it is evident that the two enriched samples, RL and RP1.5, show a substantial attenuation of unwelcome attributes without the penalties imposed by the bitter and acidic tastes present in the OL and RP3 samples.

By closely examining Table 7, which presents the mean values and model coefficients for each combination of descriptors in each product, we can identify the key characteristics. Attributes related to matrix perceptions, such as cereals, flour, oat flavor, and cooked starch, which are generally less appreciated by consumers, tend to be significantly reduced in enriched products containing raspberry powder or infusions. In contrast, these enriched products exhibit lower sweetness and higher acidity and astringency than standard oat milk. As expected, astringency is more pronounced in samples made with olive infusion and at the highest level of raspberry powder enrichment (3%).

**Figure 3:**
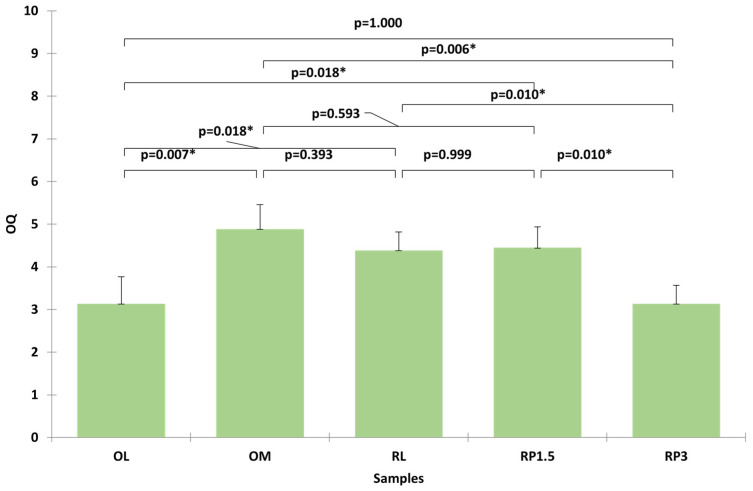
Evaluation of overall quality of samples by 25 consumers. Bars represent the mean with standard deviation. Asterisks represent statistical significance at *p* < 0.05.

The sensory profiles were further validated through an affective test involving 25 consumers, who evaluated the overall quality (OQ) of the five samples. The data, which were analyzed using the Kruskal–Wallis test, confirmed significant differences between the samples (Figure 3). OM (control) and RP1.5 received the highest ratings, with no significant difference between them. RL followed, while RP3 and OL were the least appreciated, presenting significantly lower OQ scores.

In summary, the RP1.5 formulation demonstrated a successful balance between reducing undesirable attributes and maintaining consumer acceptance. It emerged as a promising alternative to conventional oat milk, offering improved sensory characteristics without compromising overall quality.

## 4. Conclusions

In conclusion, this study demonstrated that enriching OBM with raspberry and olive leaf infusions significantly increased its total polyphenol content and antioxidant capacity compared to the control sample. However, the complete substitution of water with these infusions adversely affected the physical stability of the product. On the other hand, the addition of raspberry powder showed a stabilizing effect on the emulsion system, maintaining a low separation index while moderately enhancing the bioactive compound content. These findings suggest that, although the use of leaf infusions is an effective strategy to increase the functional properties of OBM, their impact on physical stability must be carefully considered. In this context, a combined formulation approach that integrates both infusions and powders derived from agri-food byproducts may represent a promising strategy to balance nutritional enhancement with physicochemical stability.

Further research is needed to optimize this combined approach by evaluating different ratios of infusions and powders and investigating the potential use of natural fibers or functional polysaccharides as stabilizers. Additionally, it will be essential to assess the impact of these formulations on sensory properties and consumer acceptability in order to promote the valorization of local agricultural resources and byproducts within a sustainable food production framework.

## Figures and Tables

**Table 1:** Selected descriptors used for descriptive sensory profile of OBMs.

Descriptors	
Appearance	Color intensity (CI)
Taste	Sweet (SW)
	Salty (SO)
	Acid (AC)
	Bitter (BI)
	Persistency (PE)
Flavor	Oat (OA)
	Flour (FL)
	Cooked starch (CS)
	Cardboard
	Cereal (CE)
	Red fruits (RF)
	Yeasts (YE)
Texture	Astringency (AS)
	Viscosity (VI)
Other (specify)	
Overall quality	

**Table 2:** Dry matter (DM, %), moisture content (MC, %), total polyphenol content (mg GAE mg/100 g dw), DPPH scavenging activity (TAEC mg/g dw), and ABTS scavenging activity (TE mg/g dw).

	DM	MC	TPC	DPPH	ABTS
Olive leaves	94.93 ± 0.09 a	5.07 ± 0.09 b	3195 ± 217 c	5.57 ± 0.02 c	9.54 ± 0.02 a
Raspberry leaves	94.19 ± 0.01 a	5.81 ± 0.01 b	3650 ± 17 b	5.67 ± 0.02 c	9.62 ± 0.05 a
Raspberry powder	93.67 ± 0.08 a	6.33 ± 0.08 b	2056 ± 108 d	2.76 ± 0.07 d	4.64 ± 0.12 c
Raspberry leaf infusion	0.35 ± 0.01 b	99.65 ± 0.01 a	8973 ± 62 a	21.01 ± 0.02 a	7.20 ± 0.02 b
Olive leaf infusion	0.20 ± 0.01 b	99.80 ± 0.02 a	2049 ± 29 d	16.91 ± 0.79 b	6.23 ± 0.49 b

Data are the mean of three biological replicates plus three technical replicates ± standard deviation. Different letters within columns represent significant differences (*p* ≤ 0.05) according to one-way ANOVA and Tukey’s post hoc test.

**Table 3:** Dry matter (DM, %) and moisture content (MC, %) in the different oat based milk: OM = control oat-based milk, OL = oat-based milk with the substitution of water with olive leaf extract, RL = oat-based milk with the substitution of water with raspberry leaf extract, RP 1.5 = oat-based milk enriched with 1.5% raspberry powder, and RP 3 = oat-based milk enriched with 3% raspberry powder.

	DM	MC
OM	12.28 ± 0.02	87.71 ± 0.02
OL	12.10 ± 0.01	87.89 ± 0.01
RL	12.55 ± 0.02	87.44 ± 0.02
RP 1.5	11.96 ± 0.03	88.03 ± 0.3
RP 3	12.52 ± 0.01	87.47 ± 0.01

Data are the mean of three biological replicates plus three technical replicates ± standard deviation.

**Table 4:** Total polyphenol content (TPC, GAE mg/100 g dw), DPPH (TEAC mg/g dw), and ABTS (TE mg/g dw) in the different oat-based milk: OM = control oat-based milk, OL = oat-based milk with the substitution of water with olive leaf extract, RL = oat-based milk with the substitution of water with raspberry leaf extract, RP 1.5 = oat-based milk enriched with 1.5% raspberry powder, and RP 3 = oat-based milk enriched with 3% raspberry powder.

	TPC	DPPH	ABTS
OM	385 ± 6.31 c	0.95 ± 0.05 d	4.34 ± 0.80 d
OL	555 ± 61.76 b	2.44 ± 0.15 b	12.98 ±1.67 b
RL	688 ± 77.99 a	3.68 ± 0.01 a	23.23 ± 0.67 a
RP 1.5	355.73 ± 15.21 c	1.27 ± 0.43 c	5.23 ± 0.37 c
RP 3	365.43 ± 17.96 c	1.43 ± 0.04 c	5.88 ± 0.48 c

Data are the mean of three biological replicates plus three technical replicates ± standard deviation. Different letters represent significant differences (*p* ≤ 0.05) according to one-way ANOVA and Tukey’s post hoc test.

**Table 5:** Discriminating power of sensory descriptors based on ANOVA test values and *p*-values.

Descriptors	Test Values	*p*-Values
OA	7.764	0.00002
FL	7.142	0.00001
CS	6.968	0.00003
CE	6.962	0.00004
SO	5.786	0.00002
AS	5.519	0.00001
SW	2.439	0.00701
BI	2.341	0.01020
PE	0.171	0.43210
VI	−0.540	0.70601

**Table 6:** Adjusted mean values of sensory descriptors across the different oat milk samples. Colours indicate a significant positive effect (blue) and a significant negative effect (red).

Sample	CE	FL	OA	CS	SW	VI	PE	SO	AS	BI
OM	4.56	5.25	4.625	4.62	3.75	3.18	4.00	1.81	1.87	2.25
RP 3	2.50	2.93	3.68	2.18	2.31	3.25	4.31	3.87	4.00	2.75
RP 1.5	1.81	2.50	2.37	2.68	2.75	3.00	4.18	2.75	2.65	2.31
RL	0.75	1.25	1.25	1.75	2.00	3.00	3.75	2.12	2.75	3.37
OL	1.25	2.00	1.37	1.75	2.00	3.00	3.62	2.12	4.18	3.65

**Table 7:** Model coefficients, estimated means, and *p*-values for each product–descriptor combination.

	OL			OM			RL			RP1.5			RP3		
Attributes	Coefficient	Estimated Mean	*p*	Coefficient	Estimated Mean	*p*	Coefficient	Estimated Mean	*p*	Coefficient	Estimated Mean	*p*	Coefficient	Estimated Mean	*p*
FL	−0.79	2.00	0.00	2.46	5.25	<0.0001	−1.54	1.25	<0.0001	−0.29	2.50	0.12	0.15	2.94	0.41
SW	−0.56	2.00	0.08	1.19	3.75	0.00	−0.56	2.00	0.08	0.19	2.75	0.55	−0.25	2.31	0.43
BI	0.56	3.63	0.03	−0.81	2.25	0.00	0.31	3.38	0.21	0.25	3.31	0.32	−0.31	2.75	0.21
SO	−0.41	2.13	0.01	−0.73	1.81	<0.0001	−0.41	2.13	0.01	0.21	2.75	0.15	1.34	3.88	<0.0001
PE	−0.35	3.63	0.19	0.03	4.00	0.92	−0.23	3.75	0.39	0.21	4.19	0.42	0.34	4.31	0.20
CS	−0.85	1.75	<0.0001	2.03	4.63	<0.0001	−0.85	1.75	<0.0001	0.09	2.69	0.56	−0.41	2.19	0.01
OA	−1.26	1.38	<0.0001	1.99	4.63	<0.0001	−1.51	1.13	<0.0001	−0.26	2.38	0.09	1.05	3.69	<0.0001
CE	−0.93	1.25	<0.0001	2.39	4.56	<0.0001	−1.43	0.75	<0.0001	−0.36	1.81	0.06	0.33	2.50	0.09
AS	1.10	4.19	<0.0001	−1.21	1.88	<0.0001	−0.34	2.75	0.08	−0.46	2.63	0.02	0.91	4.00	<0.0001
VI	−0.09	3.00	0.56	0.10	3.19	0.50	−0.09	3.00	0.56	−0.09	3.00	0.56	0.16	3.25	0.28

## Data Availability

The original contributions presented in the study are included in the article, further inquiries can be directed to the corresponding author.

## References

[1] Munekata P.E.S., Domínguez R., Budaraju S., Roselló-Soto E., Barba F.J., Mallikarjunan K., Roohinejad S., Lorenzo J.M. (2020). Effect of Innovative Food Processing Technologies on the Physicochemical and Nutritional Properties and Quality of Non-Dairy Plant-Based Beverages. Foods.

[2] Global Market Insights (2024). Plant Milk Market Size—By Source (Soy, Almond, Oat, Rice, Coconut), Distribution Channel (Mainstream Stores, Specialty Stores), Formulation (Unsweetened & Sweetened), Packaging (Carton Packaging, Bottles, Pouches) & Forecast, 2024–2032.

[3] Ramsing R., Santo R., Kim B.F., Altema-Johnson D., Wooden A., Chang K.B., Semba R.D., Love D.C. (2023). Dairy and Plant-Based Milks: Implications for Nutrition and Planetary Health. Curr. Environ. Health Rep..

[4] Sethi S., Tyagi S.K., Anurag R.K. (2016). Plant-Based Milk Alternatives an Emerging Segment of Functional Beverages: A Review. J. Food Sci. Technol..

[5] Reyes-Jurado F., Soto-Reyes N., Dávila-Rodríguez M., Lorenzo-Leal A.C., Jiménez-Munguía M.T., Mani-López E., López-Malo A. (2023). Plant-Based Milk Alternatives: Types, Processes, Benefits, and Characteristics. Food Rev. Int..

[6] Krampe C., Fridman A. (2022). Oatly, a Serious ‘Problem’ for the Dairy Industry? A Case Study. Int. Food Agribus. Manag. Rev..

[7] Bocchi S., Rocchetti G., Elli M., Lucini L., Lim C.Y., Morelli L. (2021). The Combined Effect of Fermentation of Lactic Acid Bacteria and in Vitro Digestion on Metabolomic and Oligosaccharide Profile of Oat Beverage. Food Res. Int..

[8] Xiong Y., Zhang P., Warner R.D., Shen S., Fang Z. (2022). Cereal Grain-Based Functional Beverages: From Cereal Grain Bioactive Phytochemicals to Beverage Processing Technologies, Health Benefits and Product Features. Crit. Rev. Food Sci. Nutr..

[9] Geburt K., Albrecht E.H., Pointke M., Pawelzik E., Gerken M., Traulsen I. (2022). A Comparative Analysis of Plant-Based Milk Alternatives Part 2: Environmental Impacts. Sustainability.

[10] Yu Y., Li X., Zhang J., Li X., Wang J., Sun B. (2023). Oat Milk Analogue versus Traditional Milk: Comprehensive Evaluation of Scientific Evidence for Processing Techniques and Health Effects. Food Chem. X.

[11] Vashisht P., Sharma A., Awasti N., Wason S., Singh L., Sharma S., Charles A.P.R., Sharma S., Gill A., Khattra A.K. (2024). Comparative Review of Nutri-Functional and Sensorial Properties, Health Benefits and Environmental Impact of Dairy (Bovine Milk) and Plant-Based Milk (Soy, Almond, and Oat Milk). Food Humanit..

[12] Cui L., Zhao J., Fan L., Ma A., Zhong F., Zhou S., Hou D. (2025). Stability and Sensory Quality of Plant-Based Milk Substitutes Made from 36 Different Oat Varieties: Relation with Oat Kernels Attributes. J. Cereal Sci..

[13] Alsado C., Lopez-Aldana L., Chen L., Wismer W. (2023). Consumer Perception and Sensory Drivers of Liking of Fortified Oat Milks. Foods.

[14] Veneziani G., Novelli E., Esposto S., Taticchi A., Servili M. (2017). Applications of Recovered Bioactive Compounds in Food Products. Olive Mil. Waste: Recent. Advances for Sustainable Management.

[15] Mirabella N., Castellani V., Sala S. (2014). Current Options for the Valorization of Food Manufacturing Waste: A Review. J. Clean. Prod..

[16] da Silva L.R., Velasco J.I., Fakhouri F.M. (2023). Use of Rice on the Development of Plant-Based Milk with Antioxidant Properties: From Raw Material to Residue. LWT.

[17] Shori A.B., Al Zahrani A.J. (2022). Non-Dairy Plant-Based Milk Products as Alternatives to Conventional Dairy Products for Delivering Probiotics. Food Sci. Technol..

[18] Zhou S., Jia Q., Cui L., Dai Y., Li R., Tang J., Lu J. (2023). Physical–Chemical and Sensory Quality of Oat Milk Produced Using Different Cultivars. Foods.

[19] Cereals & Grains Association (1999). AACC International Method 44-15.02. Moisture—Air-Oven Methods. AACC Approved Methods of Analysis.

[20] Welch R.W. (2011). Nutrient Composition and Nutritional Quality of Oats and Comparisons with Other Cereals. Oats: Chemistry and Technology.

[21] De Santis D., Ferri S., Rossi A., Frisoni R., Turchetti G. (2022). By-Product of Raspberry Juice as a Functional Ingredient: Effects on the Properties and Qualitative Characteristics of Enriched Pasta. Int. J. Food Sci. Technol..

[22] De Santis D., Ferri S., Rossi A., Frisoni R., Modesti M. (2024). Shortbread Cookies Reformulation by Raspberry Powder Enrichment: Functional and Sensory Aspects. Int. J. Food Sci. Technol..

[23] Li R., Cao H., Wang Y., Song H., Huang K., Zhang Y., Sun Q., Sun Z., Guan X. (2023). Improving Physicochemical Stability of Highland Barley-Based Milk by the Addition of Endogenous β-Glucan. Food Hydrocoll..

[24] Singleton V.L., Rossi J.A. (1965). Colorimetry of Total Phenolics with Phosphomolybdic-Phosphotungstic Acid Reagents. Am. J. Enol. Vitic..

[25] Xiao F., Xu T., Lu B., Liu R. (2020). Guidelines for Antioxidant Assays for Food Components. Food Front..

[26] Re R., Pellegrini N., Proteggente A., Pannala A., Yang M., Rice-Evans C. (1999). Antioxidant Activity Applying an Improved ABTS Radical Cation Decolorization Assay. Free Radic. Biol. Med..

[27] (1993). Sensory Analysis—General Guidance for the Selection, Training and Monitoring of Selected Assessors and Expert Sensory Assessors—Part 1: Selected Assessors.

[28] (2008). Sensory Analysis—General Guidance for the Selection, Training and Monitoring of Assessors—Part 2: Expert Sensory Assessors.

[29] ISO (International Organization for Standardization) (1988). Sensory Analysis—General Guidance for the Design of Test Rooms.

[30] ISO (International Organization for Standardization) (2003). Sensory Analysis—Methodology—General Guidance for Establishing a Sensory Profile.

[31] De Santis D., Moresi M., Cimini A. (2020). Effect of β-Glucan Enrichment on the Sensory Properties of Fresh Egg White Pasta. LWT.

[32] Yemmen M., Landolsi A., Ben Hamida J., Mégraud F., Trabelsi Ayadi M. (2017). Antioxidant Activities, Anticancer Activity and Polyphenolics Profile, of Leaf, Fruit and Stem Extracts of Pistacia Lentiscus from Tunisia. Cell Mol. Biol..

[33] Lu Y., Du Y., Qin X., Wu H., Huang Y., Cheng Y., Wei Y. (2019). Comprehensive Evaluation of Effective Polyphenols in Apple Leaves and Their Combinatory Antioxidant and Neuroprotective Activities. Ind. Crops Prod..

[34] Li C., Feng J., Huang W.-Y., An X.-T. (2013). Composition of Polyphenols and Antioxidant Activity of Rabbiteye Blueberry (Vaccinium Ashei) in Nanjing. J. Agric. Food Chem..

[35] Ren X., Yang Y., Liu Q., Wang Y., Jin Z., Jiao A. (2023). Effects of Enzymatic Extrusion on the Structure and Physicochemical Properties of Oat Flour and Its Application in Oat Milk Production. Int. J. Food Sci. Technol..

[36] Cheng G., Xue Y., Li Y., Xiong T. (2024). Research on the Basic Characteristics of Citrus Fiber and Its Application in PET Acidic Milk Beverages. Food Ferment. Ind..

[37] Tholstrup Sejersen M., Salomonsen T., Ipsen R., Clark R., Rolin C., Balling Engelsen S. (2007). Zeta Potential of Pectin-Stabilised Casein Aggregates in Acidified Milk Drinks. Int. Dairy J..

[38] McCarron R., Methven L., Ghawi S.K., Grahl S., Elliott R., Lignou S. (2025). The Effects of Processing Steps on Avenanthramides, Avenacosides and β-Glucan Content during the Production of Oat-Based Milk Alternatives. Food Chem. Adv..

[39] Escobar-Sáez D., Montero-Jiménez L., García-Herrera P., Sánchez-Mata M.C. (2022). Plant-Based Drinks for Vegetarian or Vegan Toddlers: Nutritional Evaluation of Commercial Products, and Review of Health Benefits and Potential Concerns. Food Res. Int..

[40] Meydani M. (2009). Potential Health Benefits of Avenanthramides of Oats. Nutr. Rev..

[41] Garjonyte R., Budiene J., Labanauskas L., Judzentiene A. (2022). In Vitro Antioxidant and Prooxidant Activities of Red Raspberry (*Rubus idaeus* L.) Stem Extracts. Molecules.

[42] Lebedev V.G., Lebedeva T.N., Vidyagina E.O., Sorokopudov V.N., Popova A.A., Shestibratov K.A. (2022). Relationship between Phenolic Compounds and Antioxidant Activity in Berries and Leaves of Raspberry Genotypes and Their Genotyping by SSR Markers. Antioxidants.

[43] Kotuła M., Kapusta-Duch J., Smoleń S., Doskočil I. (2022). Phytochemical Composition of the Fruits and Leaves of Raspberries (*Rubus idaeus* L.)—Conventional vs. Organic and Those Wild Grown. Appl. Sci..

[44] Umeno A., Takashima M., Murotomi K., Nakajima Y., Koike T., Matsuo T., Yoshida Y. (2015). Radical-Scavenging Activity and Antioxidative Effects of Olive Leaf Components Oleuropein and Hydroxytyrosol in Comparison with Homovanillic Alcohol. J. Oleo Sci..

[45] Chwil M., Matraszek-Gawron R., Kostryco M., Różańska-Boczula M. (2023). Nutritionally Important Pro-Health Active Ingredients and Antioxidant Properties of Fruits and Fruit Juice of Selected Biennial Fruiting *Rubus idaeus* L. Cultivars. Pharmaceuticals.

